# Views of General Practitioners on Indoor Environmental Health Risks in the Perinatal Period

**DOI:** 10.3389/fmed.2015.00032

**Published:** 2015-05-18

**Authors:** Gladys Ibanez, Jehan Zabar, Jean-Sébastien Cadwallader, Claire Rondet, Matthias Lochard, Anne Marie Magnier

**Affiliations:** ^1^Department of General Practice, Faculty of Medicine Pierre and Marie Curie, Sorbonne Universités, UPMC Univ Paris 06, Paris, France; ^2^SFTG Recherche, Société de Formation Thérapeutique du Généraliste Recherche, Paris, France; ^3^Department of General Practice, University of Tours, Paris, France; ^4^ICF Environnement, Gennevilliers, France

**Keywords:** primary care, prevention, respiratory diseases, family health, child and adolescent development, qualitative research/study

## Abstract

**Background:**

Home is generally perceived as a safety place, whereas the concentration of pollutants, influenced not only by external pollution but also by human activities, the presence of domestic animals, construction and furniture materials, are sometimes greater than outside.

**Objectives:**

The aim of this study is to determine the general practitioners’ (GPs) views on indoor environmental health risks in the perinatal period.

**Methods:**

Four semi-structured focus group with 31 GPs were conducted in two French departments in November 2009, February, March, and April 2010. The focus group meetings were analyzed using a general thematic analysis.

**Results:**

Perinatal care is a special health issue and a time of privileged sensitization. The attitude of health risks are well known in the case of “traditionally” toxic substances. In the case of “emerging” environmental exposure, these attitudes depend on the knowledge, beliefs, and experience specific to each practitioner. GPs are acquiring a new role in the field of environmental health, while at the same time coming to grips with its own strengths and limitations. The implementation of prevention depends on factors, which are not only specific to the practitioner but also related to the parents and the organization of the medical practice.

**Conclusion:**

The sensitization of GPs to environmental medicine, promotion of eco-citizen education, development of research, and the distribution of information are some of the means which need to be implemented to prevent harmful exposure of the infant.

## Introduction

The fetal programing hypothesis suggests that human health and development have their origin in early life (1, 2). Central to these hypotheses is the interdependence of developmental influences, either genetic or environmental. Pregnancy and the early postnatal period are times of great vulnerability.

Our way of life leads us to spend approximately 65–80% of our time inside various premises (domestic, work, public transportation, leisure, or public premises) (3, 4). As opposed to external pollution, which is publicized by pollution peaks and is addressed by numerous laws and regulations, air pollution in the interior is relatively poorly understood by the public at large, and only a small number of guideline values are proposed, concerning the main forms of indoor pollution (5–7). Ninety-two percent of French citizens believe that the home is a place where one is protected, whereas the concentration of pollutants, influenced not only by external pollution but also by human activities (do-it-yourself activities, housework, use of combustion appliances), the presence of domestic animals, construction and furniture materials, are sometimes greater (8–10).

Several scientific studies have recently revealed the health consequences of indoor pollution, the effects of which are particularly conspicuous during perinatal period. The main effects described in the literature, for the fetus or the young child, were the occurrence of slow intra-uterine growth, preterm birth, bronchiolitis, allergies, and asthma in infants, ear–nose-and-throat symptoms (chronic coughing, rhinitis, etc.,), or neuro-behavioral disorders in older children (11–18). In parallel, the associations between low socioeconomic status (SES) and adverse pregnancy outcomes are well documented in perinatal research (19). Adverse effects of environmental exposure can act through oxidative stress, inflammation, and/or endocrine disruption to promote developmental toxicity and adverse perinatal health (20).

The views of public health actors, such as general practitioners (GPs) on indoor environmental health risks in the perinatal period, remain unexplored. This analysis is interesting for several reasons: on the one hand, these actors regularly monitor infants and pregnant women (21); on the other hand, they have an important role in the screening, diagnosis, and monitoring of environmental pathologies (22–24). The main purpose of the present study is thus to explore these views. Secondary objectives included the way in which they assume their role in terms of environmental health and the identification of factors affecting the implementation of prevention in physician’s offices.

## Materials and Methods

### Type of study and data collection

This study was made on the basis of a qualitative observational study. The focus group method was retained in order to explore doctors’ opinions, beliefs, concerns, and ensure interactivity (25–27). Four focus groups took place in two French departments (75 -Paris- and 95 -Val d’Oise-) in November 2009, February, March, and April 2010. Each focus group comprised 7–8 GPs and lasted approximately 120 min. A doctor volunteer (Jehan Zabar) compiled a list of various physicians who worked in the two departments (randomly selected using the telephone book). The doctor volunteer contacted by phone each person listed and sought their voluntary participation. A snowball approach was then employed to expand the list of potential participants (28). The groups were hosted by a moderator and an observer. All of the focus group discussions were recorded, after having obtained written consent from the participants. A third person took care of all secretarial activities. Focus groups were maintained until theoretical data saturation. As described by Glaser and Strauss, it was reached when coding of the last transcript provided no new useful data to elaborate on the theory.

### Interview guide

The interview guide was created from a review of the literature based on MEDLINE data, using the following keywords, combined in groups of two or three words: “Attitude,” “Perception,” “Opinion,” “Health attitude,” “Environment,” “Physicians family,” “Qualitative research,” and “Focus group.” Following this search, an initial interview guide was prepared, then discussed and tested during the course of several working meetings, in order to achieve a final consensual version (Data Sheet 1 in Supplementary Material).

### Data analysis

The verbatim of each sitting was individualized, rendered anonymous and fully transcribed. Two independent researchers prepared a syntactic and progressive written analysis following each interview. The verbatim was decomposed into words, sentences, expressions, or text extracts which, initially, expressed one single, identical concept: the minimal significance units (MSU). These were classed, grouped into sub-categories, and then later into categories and themes. Several corrections of the verbatim were required in order to obtain a relevant and homogeneous coding, by means of an inductive approach through a thematic content analysis (29–32). All the ideas of the focus groups were analyzed in order to construct a controlled analysis grid with the highest possible degree of reproducibility.

### Reliability of the results

Source triangulation (written, audio, and video material) and the analysis strategy were ensured by continuously comparing the data collected by two different researchers. In addition, the results were retrospectively sent to all of the participants in order to include any possible corrections, and obtain an analyzed validation of the contents. For this research, no ethical approval was obtained because this study was only observational and all data were anonymous (33).

## Results

Four focus groups were needed in order to achieve data saturation. Out of 50 doctors who were approached, a total of 31 agreed to take part in the study. The characteristics of the participants are described in Table 1. All GPs provided health care for children and women during pregnancy. Eight of them had a public practice in “child and maternal protection centers” or “family planning.” Only two of them (6%) had specific training about environmental health issues. In the analysis, three research themes were established: Perinatal care, between vulnerability and a time of privileged sensitization; sources of information, from profane perception to scientific expertise; and the role of the GP in environmental healthcare. For each of these themes, MSU extracts, sub-categories, and categories have been summarized in Tables 2–4.

**Table 1:** **Characteristics of the participants**.

Characteristics	*M* ± SD [range] or % (*n*)
**Sex**	
Masculine	42% (13)
Feminine	58% (18)
**Age** (years)	41.4 ± 12.7 [27–63]
**Parents**	
Yes	55% (17)
Yes, young children <6 years	23% (7)
**“Organic” shopping**	77% (24)
**Smokers**	
Active	6% (2)
Withdrawn	19% (6)
Non-smoker	75% (23)
**Medical area^a^**	
Urban	84% (26)
1/2 Rural	13% (4)
Rural	3% (1)
**Current practice**	
Private	74% (23)
Public^b^	26% (8)
Follow-up of pregnant women	100% (31)
**Area of practice**^c^	
Urban	97% (30)
1/2 Rural	6% (2)
Rural	6% (2)
**Professional status professionnel**	
Medical deputy	39% (12)
Permanent position or installed	61% (19)
Teachers	13% (4)
Subscribes to journals	90% (28)
Continuing medical training	90% (28)
Trainings about environmental health issues	6% (2)
Homeopathic prescriptions	35% (11)

*^a^Rural: <2000 inhabitants; semi-rural: <10,000 inhabitants; urban: ≥10,000 inhabitants*.

^b^Including “child and maternal protection centers” and “family planning.”

*^c^Two physicians had a mixed activity in an urban, semi-rural, and/or rural area*.

**Table 2:** **Theme I. Perinatality: between vulnerability and a time of privileged sensitization**.

Categories	Sub-categories	MSU
(A) A vulnerable moment in life	(A1) Mother–child relationship	*Psycho-socio-affective environment*
		*“The indoor environment: this can also be perceived as the psychic situation of the mother when she is pregnant, and when her baby has just been born. In other words the context with respect to her work, her relationship with her partner. Was this child planned or not? This is the essence of the context into which this child is born”*
		*Sensorial environment*
		*“When I think of the baby’s environment: I think of all that it comes into close contact with, the skin, the ears, what it drinks, everything it could possibly put into its mouth. Its clothes, nappies, the bath, the things it is dressed with. For the pregnant woman and her environment, it’s like an onion: the first layers, the clothes, the physical contact, the things she eats, the air she breathes: the air, tobacco, fragrances”*
	(A2) Habitat	*A fragile cocoon*
		*“That makes me think of safety.” “Social housing: this is a social factor, but it is also an environmental factor”*
		*Causes and effects: scientific knowledge and popular knowledge*
		*“I would place all forms of toxic addiction in the first line: tobacco, alcohol, but also hashish and the like, which are far more commonly consumed than one would imagine, especially by youths. Cocaine is also frequently consumed, I am not so sure that women do not consume it, even when they are pregnant, and especially in slightly “in vogue” Parisian society. Secondly, I would consider all products such as: paints, glues, aerosols; I think there is nevertheless considerable progress to be made. Thirdly, I am highly skeptical of all forms of cosmetology, especially creams, and all types of skin products which people spread onto their skin all day long. I am highly suspicious of all of these products, even though it is not very scientific, this is my personal opinion*
		*Difficulties in hierarchisation*
		*“We do not know where the toxic substances are (*…*), neither the period of exposure nor the delay, are known”*
		*“The identification of the harmful molecule* … *because everything arises from molecules, and then which is the molecule to be identified?”*

(B) A privileged moment of sensitization		*“I finally took an interest in this, because my wife became aware of this problem, at the time when she became pregnant. She became aware of everything she ingested, she had a baby in her abdomen and became inquisitive about many more things, for example she had never previously been concerned about the color of her hair. If it’s bad for the baby, perhaps it is also bad for her, but that had never been a problem until then. When she was pregnant: that became a problem”*
		*“This is a crucial time, I think it is a time when information is received, and ten times better than normally”*
		*“A woman is far more aware of these risks when she becomes pregnant”*

**Table 3:** **Theme II. Sources of information: from profane perception to scientific expertise**.

Categories	Sub-categories	MSU
(A) Mainstream press and media	(A1) Information for the “General Public”	*“I read, more for the purposes of knowing what people read, and also because that is part of the scientific noise we are continuously subjected to. On the other hand, I do not place this on the same level as that which I read in order to learn: books, the Prat magazine, or internet sites when I need the answer to a question”*
	(A2) Difficulties with the management of information	“*That brings to mind the speed with which information is conveyed. In fact, I think it travels very fast, we are nevertheless ordinary citizens as much as doctors, we are both*”
		*“For these baby bottles: first, there was a tremendous press campaign which said: all babies are going to die!”*
		*“I know there is not yet any scientific data, but the means are not made available. We are perfectly aware that there is a horrendous level of industrial “lobbying” which means that nothing changes, in France in particular. Other countries have forbidden the use of substances which are still used in France. For example: in all household cleaning products, I know there are constituents in France which do not exist in other countries such as Germany”*
		*“They feel 100% responsible for whatever could happen to their baby, perhaps it’s a cliché: societal pressure: you are pregnant, don’t move, don’t exert yourself, otherwise for the baby…”*

(B) Specialized medical journals	(B) Scientific information	*“Yes, an INPES brochure was edited concerning domestic pollution, explained on 3 or 4 pages. It is quite practical because the conclusion is rather simple: ventilate the house as well as you can, open the bedroom windows every morning. The risks can be drastically reduced”*
		*“There is an internet site which I use frequently, to prescribe medication for pregnant women: it is the CRAT. The reference center for teratogenic agents, which is very practical. That makes it possible to find a slightly official answer concerning what can be prescribed”*
		*“It’s quite similar to what is happening here tonight with influenza. There is so much information in every direction that we, already, find it difficult to find real sources of information, I mean reliable sources of information. Influenza is really the typical example, everyone is in the dark, it’s extremely difficult to find the correct information”*
		*“I had to enter the reference into Pubmed, then I consulted the abstract without making a full search, just the abstract, you can already get a good idea by reading the abstracts, except when there are real differences. It was the only time I made an active effort to look something up”*

(C) Scientific controversies and the evolution of knowledge		*“It nevertheless took me quite some time to understand that one of my children had asthma. I can remember at the time, those kids were given Toplexi*^®^ *at night. There were physio-pathological theories about rhinopharyngitis which led to a tight throat, and which made you cough up all sorts of things, but we didn’t think of asthma. We have indeed progressed in terms of our understanding of risks and symptoms*

**Table 4:** **Theme III. The role of the general practitioner in perinatal environmental health**.

Categories	Sub-categories	Participants quotes
(A) General prevention actor	(A1) Strengths	*“Yes, it is clearly our job. It’s the essential part of our job, because our patients will not go to consult the environmentalists to discuss that sort of thing. It is our responsibility to talk about it, because we are the ones who are in contact with people”*
	(A2) Limitations	*“As for prevention, I am quite convinced that a lot is done, but I am very doubtful of its efficiency. We are always giving out all sorts of advice in every direction, but I am not sure that it is very effective* …”
	(A3) Expectations	*“That brings to mind a problem which has become every more alarming in medicine, i.e., the lack of training in environmental risks”*
		*“Information needs to be more relevant, because at the moment it’s very vague”*
		*“What matters is also that we need systems, well references, and that we can find scientific references which provide suitable answers”*

(B) Factors influencing prevention implementation	(B1) Practitioner- related factors	*“Instinctively, the foul smell of paint seems more harmful than odorless paint”*
		*“I think that patients are now really more aware, because I have really been consulted very often concerning pollution of the interior. When I started in medicine, it was never mentioned. I always thought that I should take an interest in this topic”*
	(B2) Parent-related factors	*“I think he must select the patients with whom he discussed prevention”*
	(B3) Organization- related factors	*“I think it’s also important to prioritize things”. It’s true that if one is married with a woman who smokes, and is unable to give up smoking, I think the issue of tobacco has to be raised – that’s all”*
		*“There’s never enough time, that’s what we observe every day. I think we are immersed in a world of technical acts and prescriptions, and compulsory verification rituals. I think we must certainly miss out on many environmental questions, of many different types”*

(C) Mode of prevention implementation	(C1) The first actors concerned: the health professional and the citizen	*“We must nevertheless be convinced of what we say and the recommendations we make”*
		*“We are more or less obliged to believe what we read and to pass on the good word”*
		*“These are the concerns of mothers, but not those of doctors* …* I have not yet found evidence of any real relationship between this or that factor. Therefore, scientifically, I cannot make any statement, perhaps I am wrong”*
	(C2) At the medical practice	*“For a pregnant woman, only specifically adapted prevention can be implemented. You cannot discuss everything all at once”*
		*“I find it embarrassing to give advice to certain patients and not to others. I mean that if I consider there is a risk at a certain moment, then the risk is the same for everyone (*…*)”*
	(C3) Toward a new medical paradigm: a reasonable principle of precaution	*“The principle of precaution is extremely important, I think patients need to be reassured. People are so anxious, that we spend more time reassuring young mothers than informing them”*
		*“We try to apply the principle of precaution when we don’t know”*
		*“Stop worrying, simply aerate your house. It’s a matter of common sense and it can only be beneficial”*

### Perinatal care: Between vulnerability and a time of privileged sensitization

The psycho-socio-affective, sensorial, and nutritional environments of the infant have been described as potential sources of vulnerability. The habitat was the image of a fragile cocoon. More generally, living spaces were assimilated with bubbles of well-being and threat. Indeed, the GPs evoked the paradox of a healthy habitat, not only a place of refuge but also a place leading to multiple forms of exposure. Being on the one hand, conscious of the vulnerability of pregnant women and infants, and on the other hand, solicited by the patients’ questions, GPs have observed a growing interest in pollution of the interior.

General practitioners observed a change in the perception of risks during pregnancy, and noticed behavioral changes in the interests of the infant’s well-being. The term “fusion” between mother and child was used. Parents were “more receptive” to preventive messages. In addition, the doctors noticed a growing interest in environmental subjects, and pollution of the interior, in particular (skin creams, plastic baby bottles, construction work in the child’s bedroom, etc.). This period thus appeared as “favorable in terms of prevention” and represented a likely “issue in terms of public health.”

Concerning indoor air pollutants, their harmful effects on the health of a child were commonly admitted for tobacco, alcohol, carbon monoxide, and lead. However, pollutants related to emerging environmental medical issues were more frequently debated (volatile organic compounds, mold, formaldehyde, particles, mites, etc.). There was a tendency for younger doctors to give more credit to these “new pollutants” than older doctors. The common attitudes of pathologies were dominated mainly by allergic, respiratory and skin pathologies. However, their multi-factorial origin, dose-dependent factors, unknown duration of exposure, and delay until any deleterious effects can be detected, were cited as some of the numerous obstacles confronting the evaluation of risk. Occasionally, the participants discussed their uncertainties with respect to the reality of risk, and were mainly concerned by fashionable ideas (electromagnetic fields from telephones or microwave devices, high-voltage lines, etc.).

### Sources of information: From profane perception to scientific expertise

From profane perception to scientific expertise, the GPs established a table of the advantages and drawbacks of sources of information such as media and mainstream press and compared them with more specialized sources of medial information.

Concerning these environmental issues, the press and media for the “public at large” represent widely used and appreciated sources of information, even though some reservations were expressed, mainly concerning the difficulty of managing information: speed of dissemination, strategies of dramatization or understatement, and a guilt inflicting society. Indeed, all of the participants emphasized the excessive pressure of a guilt inflicting society, which projects the symbolic image of a perfect mother. The quest for absolute security and the extreme sensitivity to any breach of children’s health were described as habits, which lead to difficulties. Therefore, access to this type of information does not result from any specific research, but represents a “passive” measure corresponding to “scientific noise.”

Scientific journals have remained the reference for health professionals, in particular, in the case of “active” research on any particular health topic. However, doctors have remained critical with respect to this type of information, in view of scientific progress and the evolution of knowledge.

### The role of the doctor in environmental health

Doctors (pediatricians, obstetricians, or GPs) have been described as the main actors in preventive environmental health. Despite the occasionally quoted presence of medical indoor environment counselor (MIEC), the lack of doctors specialized in environmental health has placed them at the heart of the prevention system. However, several difficulties such as the small number of home visits, the lack of means available for “risk evaluation,” and prevention, which was already “difficult” to implement during this period, inevitably limited the scope and contributed to a sentiment of powerlessness.

The main expectations were related to the follow-up of their actions by scientific representatives, social workers, or the media, thereby favoring eco-citizen education. The reinforcement of professional medical training as well as the accessibility to “practical scientific information” were also quoted as being indispensible.

The implementation of prevention was found to depend on factors of risk acceptability specific to doctors, parents, and organizational factors. The factors related to doctors were their professional and personal beliefs and experiences. The factors related to the parents were their personality profile: “organic” or “anxious,” as well as their socioeconomic conditions (favoring exposure to indoor air pollution and limiting the actions required to improve the habitat). Finally, the time required and the need for a priority in the prevention messages were the organizationally limiting factors. From the “feeling of danger” to “preventive action,” the confrontation of opinions and attitudes sometimes revealed divergences between the doctors’ approach, as either a “citizen” or a “health professional.” Some doctors’ spoke of the need to be “convinced” themselves of the potential effects of a certain type of pollution before entering into a discussion with their patients. Others, on the contrary, distinguished between their convictions and passed on preventive messages as soon as any suspicion was raised concerning a particular pollutant.

The concrete modalities for the implementation of prevention in the medical practice were finally debated: information available in the “waiting room” or addressed “during the consultation”; “individual” prevention when requested by patients, or “generalized” prevention. The premises where the information was supplied was one of themes discussed. In general, the doctors who were questioned preferred the lack of personalization of the waiting room, but emphasized the strong risk of missing the target: patients exposed to a high level of pollution in the home could be socially disadvantaged and less inclined to read the dedicated prevention guides in the waiting room. They recommended the interactive and beneficial nature of the consultation in the doctor’s office, but feared a saturation of preventive messages during pregnancy or the neonatal period, poorly perceived intrusion into the patient’s privacy, or inappropriate anxiogenic reactions. Depending on the groups, adapted prevention could be carried out “individually” when requested by the parents, in the medical practice, or could be delivered in a more “generalized” fashion to a targeted population.

In conclusion, the GPs agreed on the idea of applying the paradigm of a reasonable principle of precaution. Indeed, they indicated their willingness to be pragmatic and apply their common sense.

## Discussion

### Summary of results

Perinatality is a specific sanitary issue, and a time of privileged sensitization. Health risks for the infant and the fetus are well known for commonly recognized toxic substances. In the case of more recently discovered forms of environmental exposure, these have depended on the doctor’s own specific knowledge, culture, and experience. The media and medical journals appeared to be the main sources of information. GPs are now adopting a new role in the field of environmental health, while at the same time remaining aware of his/her strengths and limitations. The implementation of prevention in the medical practice then depends on several factors specific to the doctor, and also on factors related to the parents and the organization of healthcare. Improved accommodation of risk considerations in the medical practice will require further evaluation, accompanied by practical actions on the part of health authorities and learned societies.

### The strength and limitations of the present study

To the best of our knowledge, no other studies have explored the views of doctors regarding environmental health risks during the perinatal period. The qualitative method was chosen in view of its capacity to bring out new ideas in terms of social attitudes, to evaluate expectations, and to explore the behavior of populations. The diversity of GPs, in particular, with respect to their difference in age, mode, and the location of their practice, and personal and professional experience, was favorable for the development of fruitful exchanges. During the course of the interviews, an “opinion leader” sometimes stood out in each focus group, and was then contained by the moderator; another limiting effect was that of “giving a good impression,” or responding to supposed expectations: the aspect of “social desirability” was difficult to avoid. It is possible that the participants sometimes gave a response, which was expected, rather than that corresponding to their beliefs or personal convictions. This limitation is, however, partially taken into account by the focus group principle, which encouraged debate among the participants. Other limits are present in this study. Identification of GPs was not reported in the tables. These data could be of interest, for example, to make some hypotheses regarding associations between individual characteristics and perceived role in environmental field. Moreover, it would be important to indicate if the GPs had a low, medium, or high activity in pregnancy care or monitoring children because it could influence the involvement of physicians in this field. Few of them had a specific training about environmental health issues. Results concerning “sources of information” or “the role of the doctor in environmental health” could be different in other focus groups. Results of this study probably do not fully describe GPs’ sensitization about environmental health issues and perinatal issues. In two focus groups, some doctors were reluctant to confirm the reality of risk. Overall, focus group samples are usually small and purposively selected. They do not allow for generalization to larger populations. These aspects could be taken into account in further studies.

### Comparison with data found in the literature

Most studies regarding the perception of environmental risks focused on physicians’ views. Studies related to doctor’s perceptions or attitudes of sanitary risk, such as atmospheric pollution, or that related to waste and incineration, have achieved results similar to those presented here (34–36). As in the present study, Medina et al., Attané et al., and Lhuilier et al. have emphasized the difficulty of establishing a definite link between pathologies and new environmental factors, and thus of evaluating their concrete impact on health. Globally, doctors were poorly informed about the health risks in question. Doctors considered the most common sources of information, such as the media and mainstream press, to be comparatively unreliable, and responsible for biases in the perception of health risks. Scientific information was then the only legitimate form of data, in the eyes of the doctors, even if they recognized that they were often unavailable or sometimes the subject of controversy. These similar results were also encountered in the study made by Antson et al. In addition, a lack of professional training has often been revealed, in particular by Rotily et al. in their quantitative study (37, 38).

In the United States, Adams et al. examined the influence of two dimensions of the pollution exposure experience – the community context and the study’s report-back process – on participants’ perceptions of exposure (39). Participants talked extensively about outdoor pollution, but they were not well informed about indoor exposures. Authors found that participants in a low-income, largely minority community were as capable as the more-educated residents of learning from an intensive report-back study on household air and dust exposure. All participants were capable of understanding scientific ideas such as the notion of cumulative exposure. Altman et al. reported interviews conducted with women about environmental chemicals in body fluids and household air and dust (40). Participants were aware that they lived in a region with elevated rates of breast cancer, several sources of air and groundwater pollution, and a fragile ecosystem. The majority cited local contamination problems (a local military base and Superfund site, two power plants, one nuclear powered, and an extensive history of pesticide application to cranberry bogs, wetlands, and golf courses). Finally, Auffret et al. performed a French qualitative study to explore parents’ perceptions about indoor environment (41). Parents reported becoming increasingly aware of environmental risks during the perinatal period. Their behaviors changed during this period: they painted children’s rooms, purchased new furnitures, etc. Four profiles of parents have been identified from less sensitized to most pro-actives. Parents had a great trust in their GPs and they were willing to ask questions to them.

### Perspectives

Perinatal care is a special health issue because it is both a time of increased vulnerability and a time of privileged sensitization. During the regular antenatal care consultations, practitioners should systematically consider social or environmental risk factors because they are as decisive as biomedical risk factors for perinatal health (42). Social risk factors could include couple situation during pregnancy, maternal employment, and type of health insurance (43). Environmental risk factors could include tobacco smoke, renovation works in the family home to welcome the future baby (paints in the baby’s bedroom, new furnitures, other building materials such as carpets or ceiling tiles) as well as reported mold in the house. Specially, if parents or children are affected by some chronic conditions such as asthma, allergy, chronic cough, and atopic dermatitis, it would be valuable to detect potentially harmful environmental exposures. Then, some recommendations could be provided by health professionals: airing rooms regularly at home, reducing the humidity levels in the house (especially in the baby’s bedroom), preferring renovation works in the second trimester of the pregnancy to minimize pollution to the newborn. Then, GPs are frequently grappling with patients’ concerns about pollutants in housings. Recommendations could be to maintain potential exposures “as low as reasonably achievable” (ALARA principle), taking into account that effects are greater in case of a chronic exposition and in case of special vulnerability (perinatal health, chronic medical condition).

The present study allows the apprehension of social attitudes and the initiative of therapeutic education to be reconciled (44). According to Gaudreau, “taking social attitudes into account in the preparation of educative interventions would favor the development of more highly integrated health (…)” (45). Our study could thus contribute to the elaboration of research strategies and a health education campaign (46, 47). One remaining priority is the development of research in order to reduce uncertainties, and to implement regulations. The other priority of environmental health prevention is to reduce social inequalities with respect to risks. The most disadvantaged populations are also the most exposed to environmental issues (8). The harmonization of rhetoric, consolidation of medical training for poorly informed doctors during their studies, and development of networks or means for environmental monitoring could become indispensable. The MIEC, who are relatively poorly known, could make invaluable contributions (48, 49). However, their small number means that their territorial coverage is inadequate. The economic criterion is certainly a factor limiting their actions. Appropriate tools for the practice of urban medicine should be distributed to all doctors, in order to simplify screening or diagnoses. Finally, priority should be given to the practice of preventive medicine in the healthcare system, by promoting home visits, and providing eco-citizen education in order to promote an environmental culture. All of these proposals are designed to assist the GP in taking a greater interest in environmental health.

## Author Contributions

GI, JC, and AM designed and managed all aspects of the research. JZ participated in the acquisition and interpretation of data. GI and JZ wrote the first draft of the paper and contributed to later drafts. ML and CR commented on the paper. All authors read and approved the final manuscript.

## Conflict of Interest Statement

The authors declare that the research was conducted in the absence of any commercial or financial relationships that could be construed as a potential conflict of interest.

## Supplementary Material

The Supplementary Material for this article can be found online at http://journal.frontiersin.org/article/10.3389/fmed.2015.00032
