# Supplementary material for: Views of General Practitioners on Indoor Environmental Health Risks in the Perinatal Period
Source: Front Med (Lausanne). 2015 May 18;2:32. doi: 10.3389/fmed.2015.00032 (PMC4434900; doi:10.3389/fmed.2015.00032)
Supplement: Supplementary file 1 [file Data_Sheet_1.PDF]

Appendix : Interview guide

**Q1. What does the expression "Environment of the pregnant women and the infant" call to mind for you?**<br><br>**Q2. In your opinion, what are the sources of indoor pollution, which could have an impact on the health of the pregnant woman and the infant? Could you rank these pollutants in terms of the health risk they represent?**<br><br>**Q3. What pathologies can you identify with respect to the environment in the home of the pregnant woman and the infant?**<br><br>**Q4. What are your sources of information? What is your experience in relation to indoor pollution for the case of pregnant women and infants?**<br><br>**Q5. Do you think the general practitioner can be helpful for young or future parents, in terms of environmental health?**
